# Development of anti-infliximab antibody is associated with reduced efficacy and infusion reaction in Behçet's disease with uveitis

**DOI:** 10.1186/1546-0096-13-S1-P166

**Published:** 2015-09-28

**Authors:** Y Ishigatsubo, M Takeno, Y Kirino, N Mizuki

**Affiliations:** 1 Yokohama City University Graduate School of Medicine, Department of Internal Medicine and Clinical Immunology, Yokohama, Japan; 2 Yokosuka City Hospital, Rheumatic Diseases Center, Yokosuka, Japan; 3 Behcet's Disease Research Committe, Yokohama, Japan; 4 Nihon Medical School Graduate School of Medicine, Department of Allergy and Rheumatology, Tokyo, Japan; 5 Yokohama City University Graduate School of Medicine, Department of Ophtalmology, Yokohama, Japan

## Backgrounds

Infliximab (IFX) potently suppresses ocular attacks in Behcet's disease (BD) with uveitis, resulting in favorable long-term visual prognosis. However, about one third of the patients had ocular attacks one or two weeks before the next IFX infusion, suggesting that the efficacy of IFX depends on the concentration. This study investigates IFX trough levels and antibody toward IFX (ATI) in BD patients receiving IFX and analyzes the relationship of the pharmacokinetics with clinical efficacy.

## Objective

To investigate effects of infliximab (IFX) trough levels and antibody toward IFX (ATI) on efficacy and adverse events in Behçet's disease (BD) patients receiving IFX.

## Patients and methods

A total of 160 BD patients who were or had been receiving IFX were enrolled (male 115, age 43.2+ 12.1 y.o.) from 10 institutes in Japan. The primary target was eye in 135 patients including 8 patients who previously discontinued IFX, intestine in 18, and CNS in 7. After initial boosting, IFX was given every 8 weeks but the interval was shortened when clinical efficacy was insufficient. The trough level was measured in sera from 122 patients with uveitis when 5mg/kg of IFX was administered with 5 to 10 week interval, whereas ATI was determined by ELISA in all patients. The patient conditions at sampling were divided into the symptomatic phase when the patients had any of symptoms due to BD except oral aphthae, and the asymptomatic phase when the patients were asymptomatic. Patients were classified into 5 groups according to physicians' global assessment of IFX efficacy for uveitis; Group A: very effective, B: effective, C: insufficient, D; ineffective, and E: discontinued the IFX.

## Results

Mean serum IFX trough level was 4.4+ 4.7mg/ml in 430 samples from 122 patients. The level was significantly lower in symptomatic phase (n=73, 2.5±5.3 μg/ml) than in the asymptomatic phase (n=357, 4.9 ± 4.6μg/ml). ROC analysis determined the cut-off level was 0.93μg/ml. Patient-based analysis revealed that the trough level was lower in Group C+D (1.3±3.3μg/ml) than Group A (4.2±4.4μg/ml) and B (5.4±5.7μg/ml), whereas administration interval was significantly shorter in Group B (7.2±1.1 wk) and C+D (6.8±1.5 wk) than Group A (8.2±0.9 wk). ATI(+) was found in 18 (11.3%) of all patients. The frequency was significantly higher in Group C+D (5/10, 50%) and E (4/8, 50%) than Group A (3/75, 4%) and B (6/38, 16%). Thus, unfavorable clinical responses were associated with low trough level and positive ATI. Muitivariate analysis using logistic regression model revealed association of therapeutic failure (Group C+D and E) with female, positive ATI, and infusion reaction (IR). Moreover, ATI was strongly associated with IR. We found that shortening administration intervals restored IFX level with clinical efficacy in 2 ATI positive patients, whereas switching to adalimumab was successful in 2 patients having serious IR.

## Conclusion

ATI is associated with reduced efficacy due to decreased IFX trough level, and IR. When IFX efficacy is reduced, shortening IFX administration intervals restores clinical efficacy in most of patients but other therapeutic option such as adalimumab is necessary in a part of the patients.

